# Radiological evaluation before and after treatment with an osseointegrated bone-anchor following major limb amputation—a guide for radiologists

**DOI:** 10.1007/s00256-023-04524-z

**Published:** 2023-12-04

**Authors:** Norbert Kang, Yazan Al-Ajam, Phyllis Keen, Alexander Woollard, Hannah Steinitz, Joanna Farrant, Geoffrey Chow

**Affiliations:** 1 Department of Plastic Surgery, Royal Free Hospital, Royal Free London NHS Foundation Trust, London, UK; 2 Faculty of Medicine, Imperial College London, Sir Alexander Fleming Building, Imperial College Rd, London, UK; 3 Department of Radiology, Royal Free Hospital, Royal Free London NHS Foundation Trust, London, UK

**Keywords:** Amputation, Bone-anchor, Direct skeletal fixation, Osseointegration, Radiology

## Abstract

Osseointegrated implants have been developed to allow direct skeletal fixation of a prosthesis as an alternative to traditional socket-fitted prostheses for patients who have suffered from a major limb amputation. The implants contribute to improvements in functional outcome and quality of life and radiological evaluation plays a crucial role in pre- and post-operative assessment. This article acts as a guide for radiologists who may be tasked with providing the radiological information required by surgeons and prosthetists. We also look at the radiological appearances of complications that may arise in patients treated with an osseointegrated implant. Plain X-rays are used to screen patients who wish to undergo treatment. Limb-length X-rays are then used to measure the length of any residual bone, and comparisons can be made with the normal side (if present). From this, decisions about the likely size of the implant and the need for further amputation can be made. CT scans enable accurate assessment of the medullary cavity and cortical thickness. Post-operatively, plain X-rays form the mainstay of the routine monitoring of the bone-implant interface. Potential complications include infection, aseptic loosening, mechanical fracture of the implant and periprosthetic fracture. Infection and aseptic loosening can be seen as a lucency at the bone-implant interface which (if left untreated) can lead to loss of the implant. Implant and periprosthetic fractures are radiographically obvious. Radiologists involved in the care of patients undergoing treatment with an osseointegrated implant should become familiar with the imaging requirements so they can contribute to optimal patient outcomes.

## Introduction

**Fig. 1:**
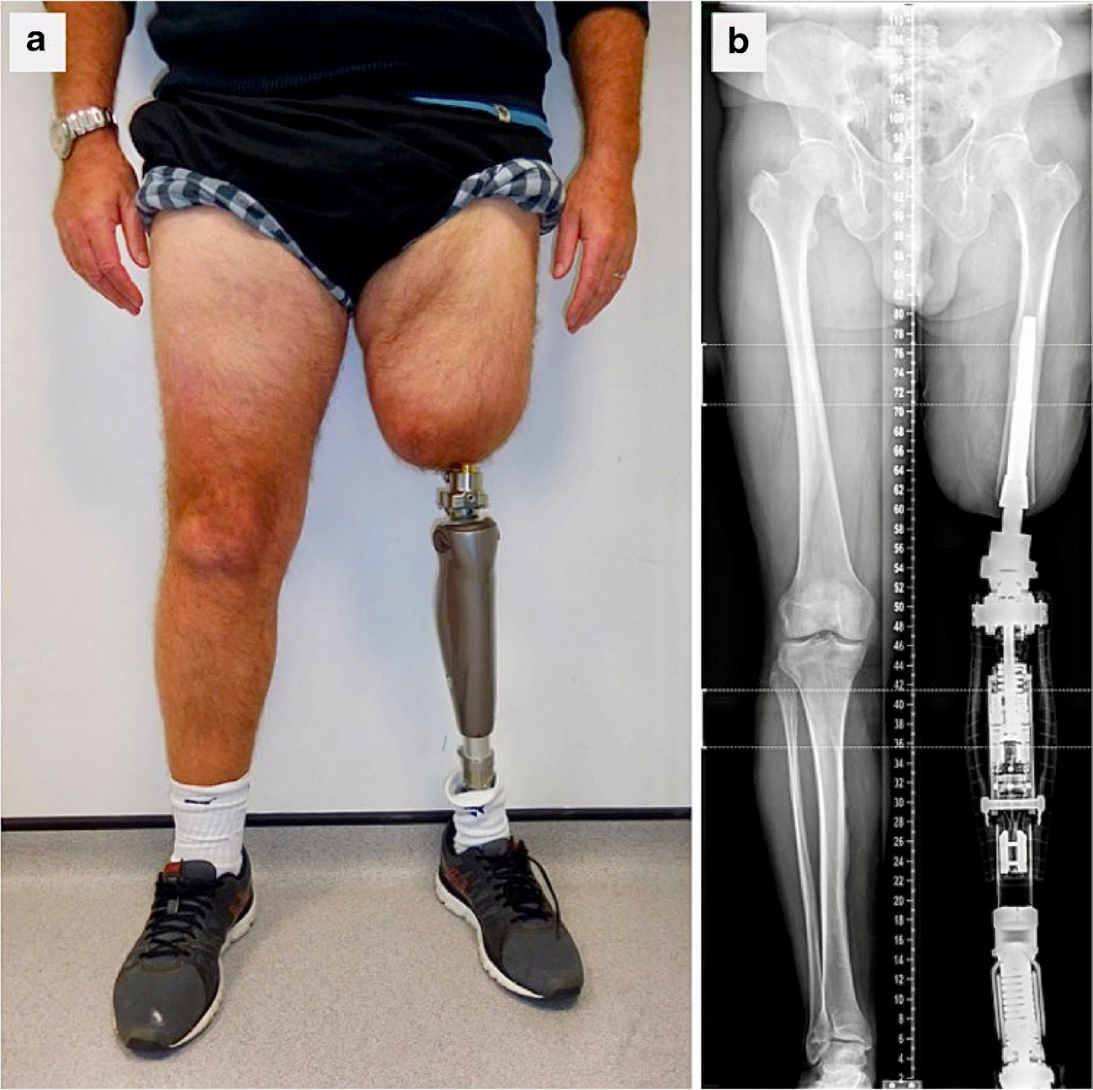
**a** Photograph and **b** leg length radiograph of a left transfemoral amputee with OPL bone-anchor in place at 3 years post-op

After amputation of a limb (either upper or lower), the most common method of reconstruction is a prosthetic device which is secured to the patient’s body using a socket and/or straps. This technique for securing a prosthesis has existed for thousands of years [1, 2]. However, its disadvantages include pain in the residual limb, skin ulceration (e.g. in the groin) and poor restoration of function [3, 4]. Such problems have spurred the development of surgical implants that enable direct fixation of the prosthesis to the patient’s residual skeleton [2, 3]. These metal implants are fixed in the bone using the principle of osseointegration which ensures that the implants do not loosen or fall out when put under load (e.g. when an artificial limb is attached) (Fig. 1). Therefore, the implants are also referred to as bone-anchored implants and the terms osseointegrated implant, bone anchor and direct skeletal fixation are often used interchangeably in the literature.

As the number of patients treated with a bone-anchor increases, so does the need to understand the natural history of these implants. Amputees who are treated with a bone-anchor should only receive treatment as part of a multi-disciplinary team that is able to look after their (often very complex) needs. Not least of these team members are radiologists who help with definitive decisions about patient suitability for implantation and also assist in monitoring the bony health of the implant, especially when there are concerns about the possibility of loosening or infection.

In this article, we present a step-by-step guide for radiologists who may be involved in the care of amputees treated with an osseointegrated bone-anchor. We highlight the specific information that surgeons and prosthetists are looking for when making pre-operative decisions about implantation. We then describe the radiological features that represent a good outcome after surgery and contrast this with red flags that suggest that the bone-anchor may be at risk and that further surgical intervention is needed.

### Types of implant

**Fig. 2:**
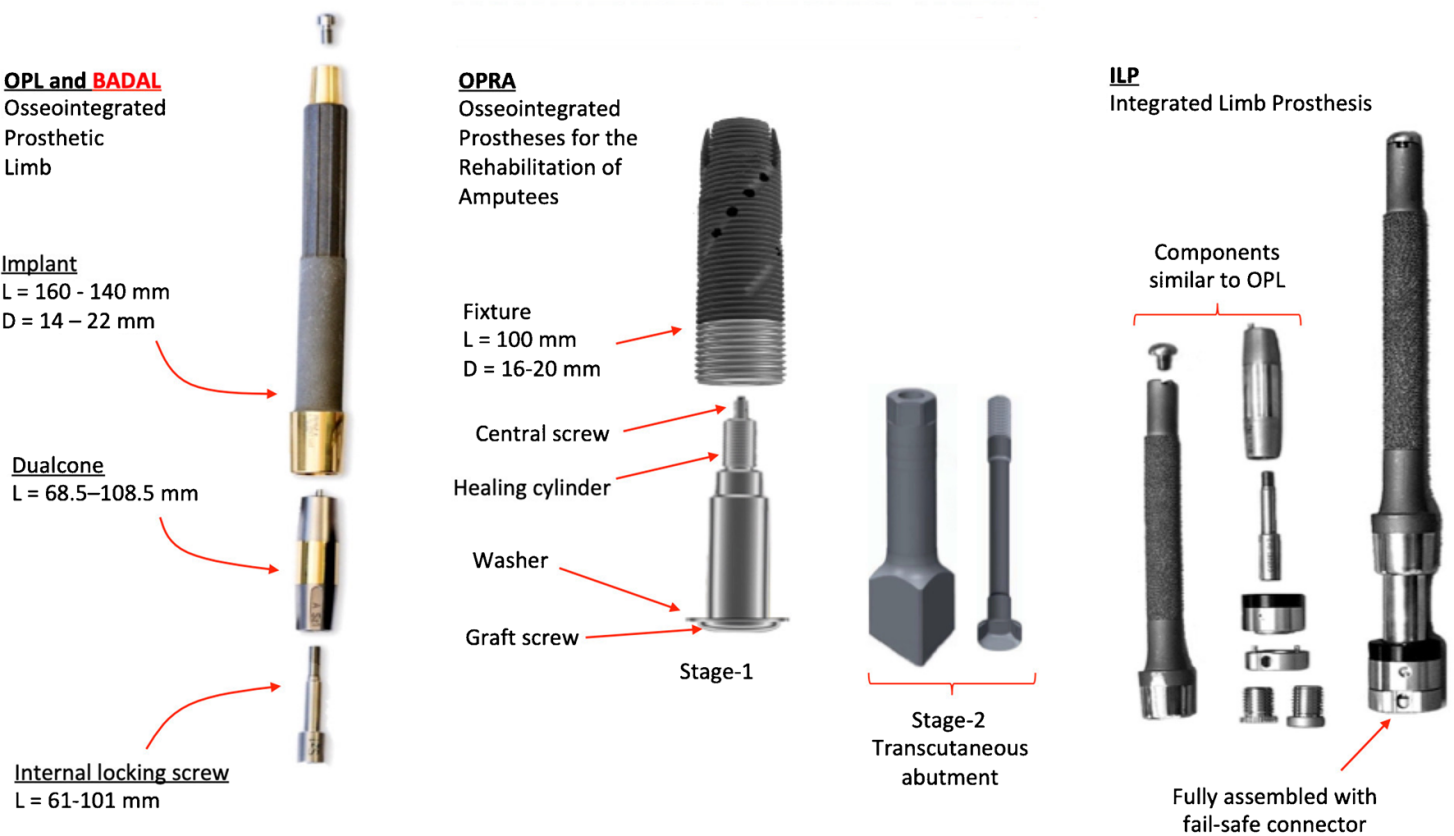
Bone-anchored implants in use across the globe. All the implants are made of titanium. **Left** The OPL was designed in Australia. **Middle **The Osseointegrated Prosthesis for Rehabilitation of Amputees was designed in Sweden.** Right** The ILP was designed in Germany

Currently, there are only two implant systems in widespread use as a bone-anchor after amputation (Fig. 2). These are the Osseointegrated Prosthetic Limb (OPL), designed in Australia [3, 5, 6], and the Osseointegrated Prosthesis for Rehabilitation of Amputees, designed in Sweden [2, 3]. These two implant types are different in terms of their shape and method of fixation in the bone, although both rely on the principle of osseointegration. The OPL implant is a press-fit design, relying on friction between the bone and the implant for primary stability. The Osseointegrated Prosthesis for Rehabilitation of Amputees implant relies on screw-fixation for primary stability.

Other bone-anchor designs exist (e.g. Integrated Limb Prosthesis (ILP) [3, 7] or bone-anchored device for amputated limbs (BADAL) [8]) or are being introduced, especially since the principles of osseointegration are becoming more widely understood. The newer implant designs share similarities with the OPL implant, and they function in the same way once placed in the bone [9]. However, the OPL implant is the design which has been used most frequently (> 1500 cases worldwide) and is therefore the one most likely to be encountered by radiologists in the future. Therefore, for the purposes of this article, we will focus on the radiological features relating to the OPL implant.

### Critical pre-operative information for prosthetists and surgeons

**Fig. 3:**
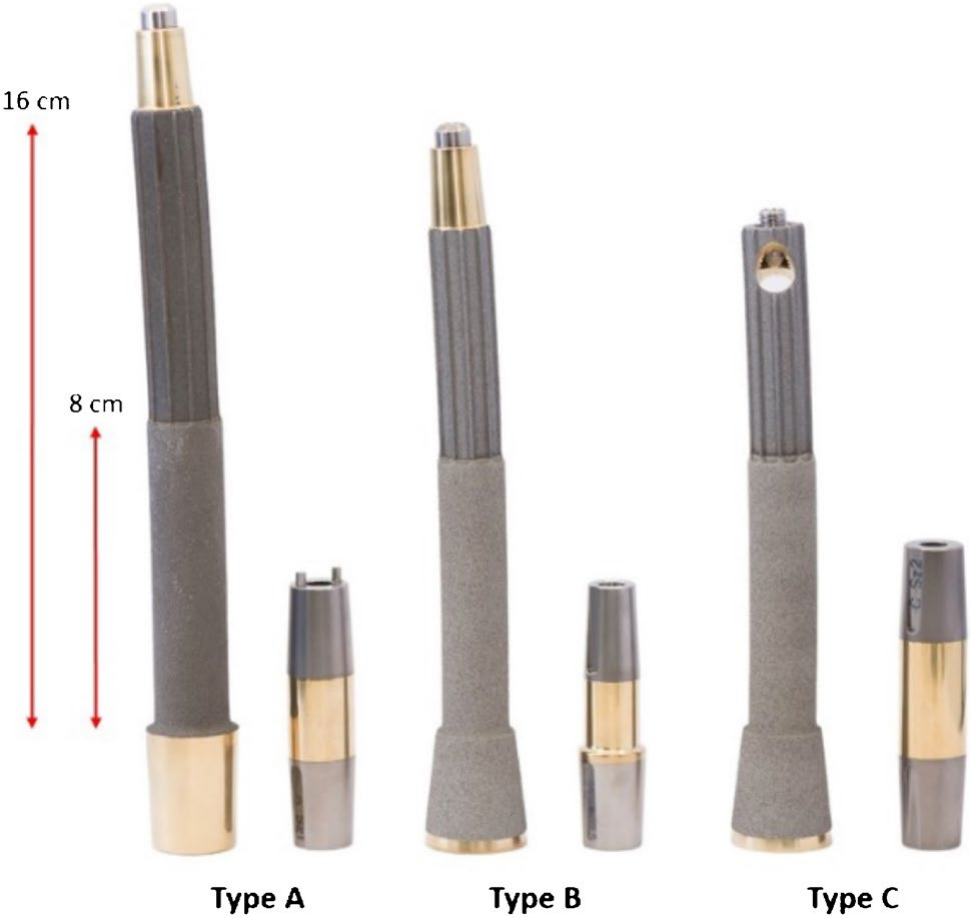
OPL implant types and implant lengths. The distal 8 cm of the implant is critical for osseointegration. The total length of the implant can be modified on a patient-to-patient basis however the minimum total intramedullary length is 10 cm. Each implant comes in a range of diameters from 14 to 22 mm

There are three types of OPL implant in current use—Type A, B and C. Of these, the most commonly used is Type A. The lengths of the different implants are indicated in Fig. 3. Each implant comes in range of diameters from 14 to 22 mm.

The critical part of the implant for osseointegration is the first 8 cm, through which the forces are transmitted from the bone to the implant. Therefore, when planning for surgery, the most important information needed by the surgeon is the following:

- The length of the residual bone (must be 10 cm or more)
- The internal diameter of the bone into which the implant will be inserted

For both of these, a planning CT scan is required. Ideally, the CT images should provide true axial and longitudinal cross-sections of the bone and multiplanar reconstruction (MPR) may be used to ensure this.

**Fig. 4:**
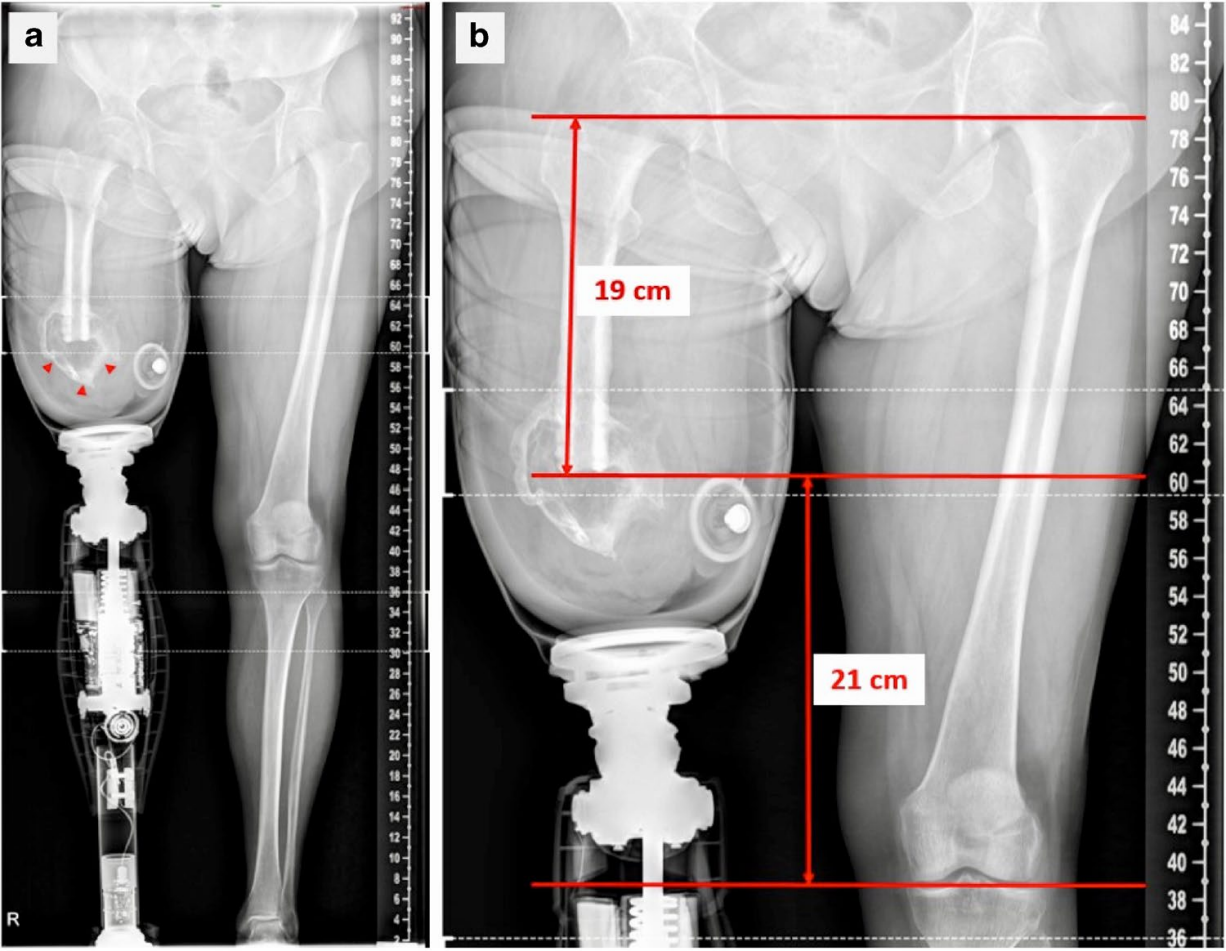
**a** AP leg-length radiographs of a right transfemoral amputee obtained standing and weight-bearing through the patient’s existing prosthetic solution. **b** The residual bone length (from amputated surface to greater trochanter) and distance from the knee joint on the intact side to the amputated surface are measured for pre-operative planning (19 and 21 cm in this example, respectively). Heterotopic ossification (a, arrowheads) is removed at the time of insertion of the bone-anchor

The surgeon and prosthetist also require pre-operative long-leg X-rays of the patient. For the lower limb, these must be obtained with the patient standing upright—ideally with their existing prosthesis attached to the limb (for lower limb amputees). This will allow measurements of their limb lengths to be obtained (Fig. 4). The critical measurements required for a transfemoral amputee are the following: distance from the amputated surface to the knee joint on the intact side (assuming a unilateral amputee) and the residual length of the amputated femur measured to the greater trochanter. The minimum distance from the end of the amputated femur to the knee joint on the normal side should be 16 cm or more. If it is less than this, the femur needs to be re-amputated to a higher level in order to accommodate the length of the typical connector components of the OPL bone-anchor. The actual connector components used can vary in height (from 6 to 9 cm depending on the connector system used). The minimum length of the residual femur to the greater trochanter must be 10 cm to accommodate the length of the implant.

**Fig. 5:**
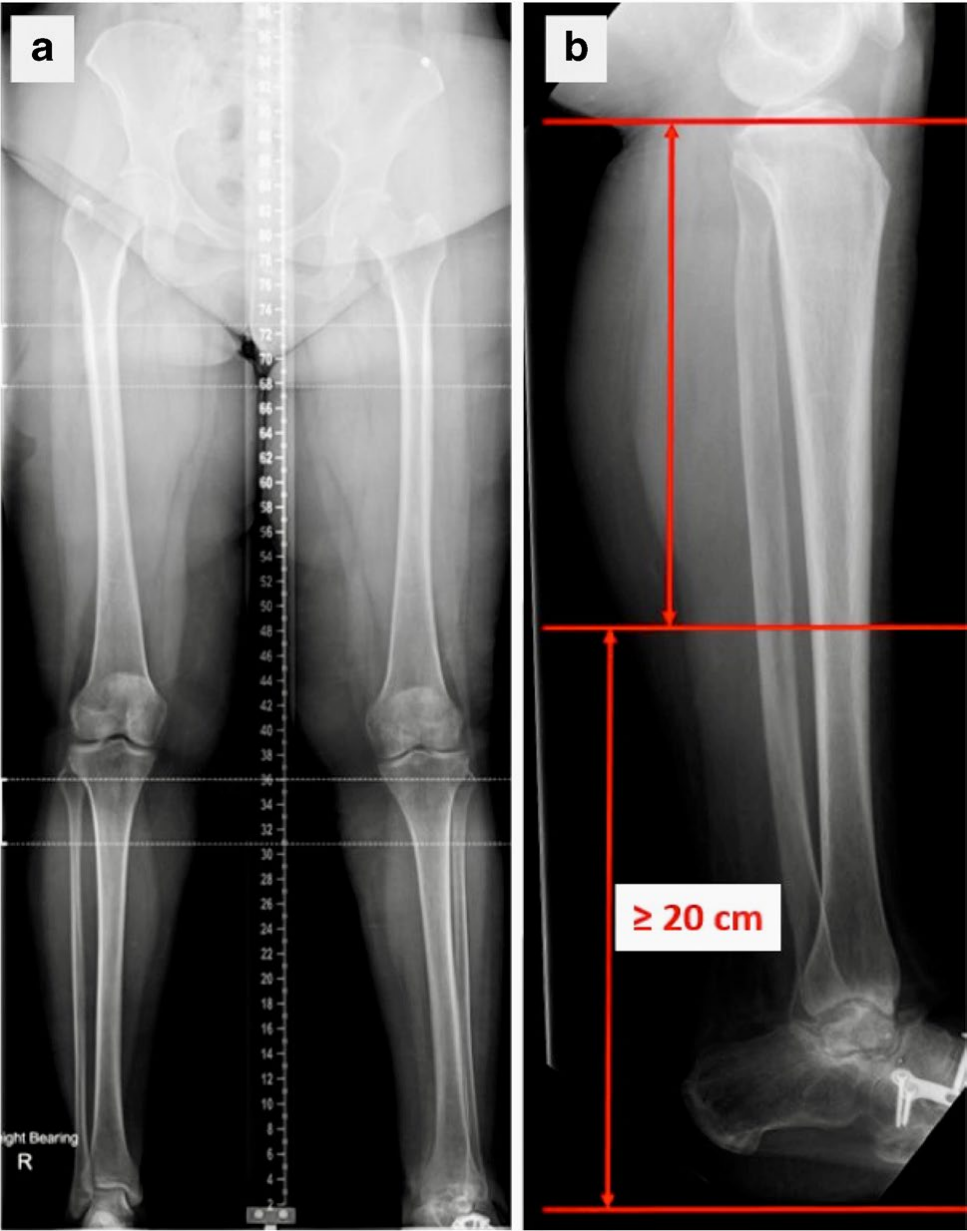
**a** AP leg length and **b** lateral pre-op standing radiographs of a patient undergoing an elective left below knee amputation. The lateral view is used to estimate the position of the isthmus of the tibia which is the best location for bone-anchor placement. The amputation should also allow a minimum distance of 20 cm from the floor to accommodate the length of the prosthetic connector components

The pre-operative measurements required for a transtibial amputee are different. Here, the bone-anchored implant should be placed at the isthmus of the tibia or just below it, as this is the point at which the fixation of the implant into the residual bone is strongest (Fig. 5). The location of this point varies from one individual to another (depending on their height), but as a general rule, it begins between 20 and 25 cm above the floor. This measurement should be obtained from a lateral view X-ray with the patient standing barefoot and fully weight-bearing on their normal limb. If they are a bilateral amputee, then an estimate must be made based on the narrowest point of the medullary canal. Once this point has been established, a minimum 20 cm from the floor is required to allow enough space for the connector components.

**Fig. 6:**
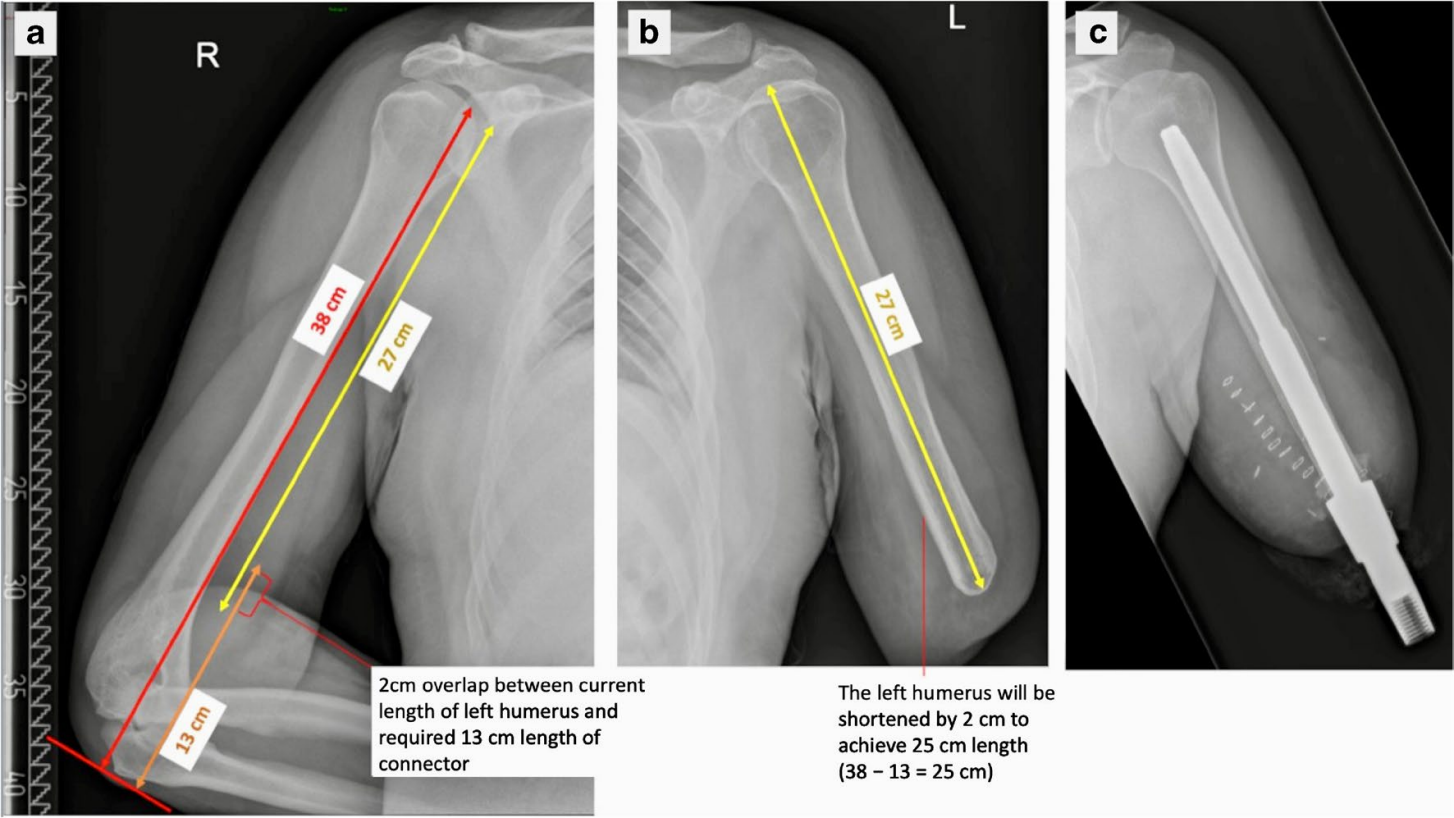
Left transhumeral amputee planning radiographs. **a** The length of the normal limb is measured from the humeral head to the level of the olecranon with the elbow flexed at 90°. In this example, it measures 38 cm. **b** The amputated humerus is also measured to the amputated surface (27 cm). A minimum distance of 13 cm is necessary to accommodate the connector components of a prosthetic limb. If the length of residual bone and connector components exceeds that of the intact limb (as in this example: 27 + 13 = 40 cm, greater than the 38 cm length of the intact limb), additional bone needs to be removed at the time of implant insertion. **c** Left transhumeral amputee of a different patient showing OPL bone-anchor in situ

The critical measurements for a transhumeral amputee are different again because the connector components for the OPL implant in the upper limb are different to those for the lower limb. X-rays of the normal limb are taken with the elbow joint flexed at 90° and resting on a table (Fig. 6). The intact humerus is measured from the humeral head to the olecranon and the residual bone length is also measured on the amputated side. A minimum difference of 13 cm is required between the intact limb length and the amputated humerus to accommodate the implant and its connector components. If the difference in lengths is less than 13 cm, additional bone needs to be re-amputated at the time of implant insertion. This ensures that the patient’s limb lengths (with the prosthesis attached) will remain equal and not compromise function or aesthetics. The minimum length of the residual humerus must also be 10 cm to accommodate the implant length.

### Role of CT scans

**Fig. 7:**
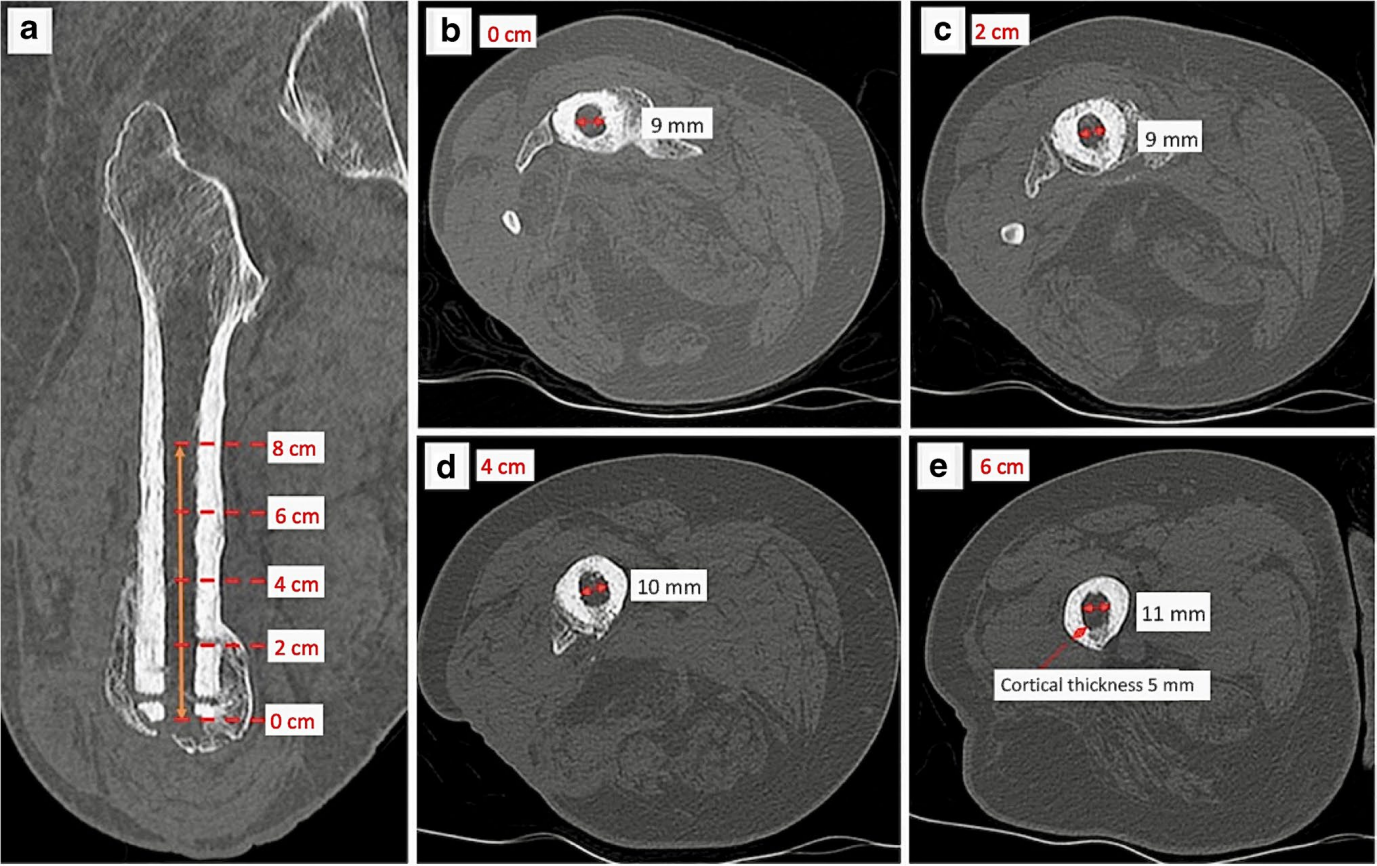
Right transfemoral amputee planning CT. **a** A coronal multiplanar reconstruction is used to evaluate four 2-cm intervals proximal to the amputated surface. **b–e** The medullary cavity diameter of the residual femur is measured in millimetres on true axial images beginning at the distal end of the femur and at four 2-cm intervals proximally up to 8 cm (i.e. equivalent to the length of the osseointegrated implant). **e** Minimum cortical thickness pre-operatively for safe implantation is 1 mm. This is measured at each segment to help with finalising decisions about the implant diameter to be used

CT scans are used for pre-operative assessment of the shape and internal diameter of the residual bone and the cortical thickness. Allowing for the characteristics of the specific implant to be used, the surgeon needs to know the diameter of the medullary canal at 1–2 cm intervals from the most distal point of the residual femur, tibia, or humerus. For a standard implant, the minimum diameter of the medullary canal is 14 mm. However, medullary diameters that are less than this can be enlarged at surgery—as long as the cortical thickness is adequate pre-operatively. Patients need a minimum of 1 mm of cortical thickness to be left after reaming of the bone to avoid problems with painful flexing of their residual bone after implantation. Custom implants with diameters less than 14 mm may also be used—especially in the humerus. An example of the measurements needed is shown in Fig. 7.

**Fig. 8:**
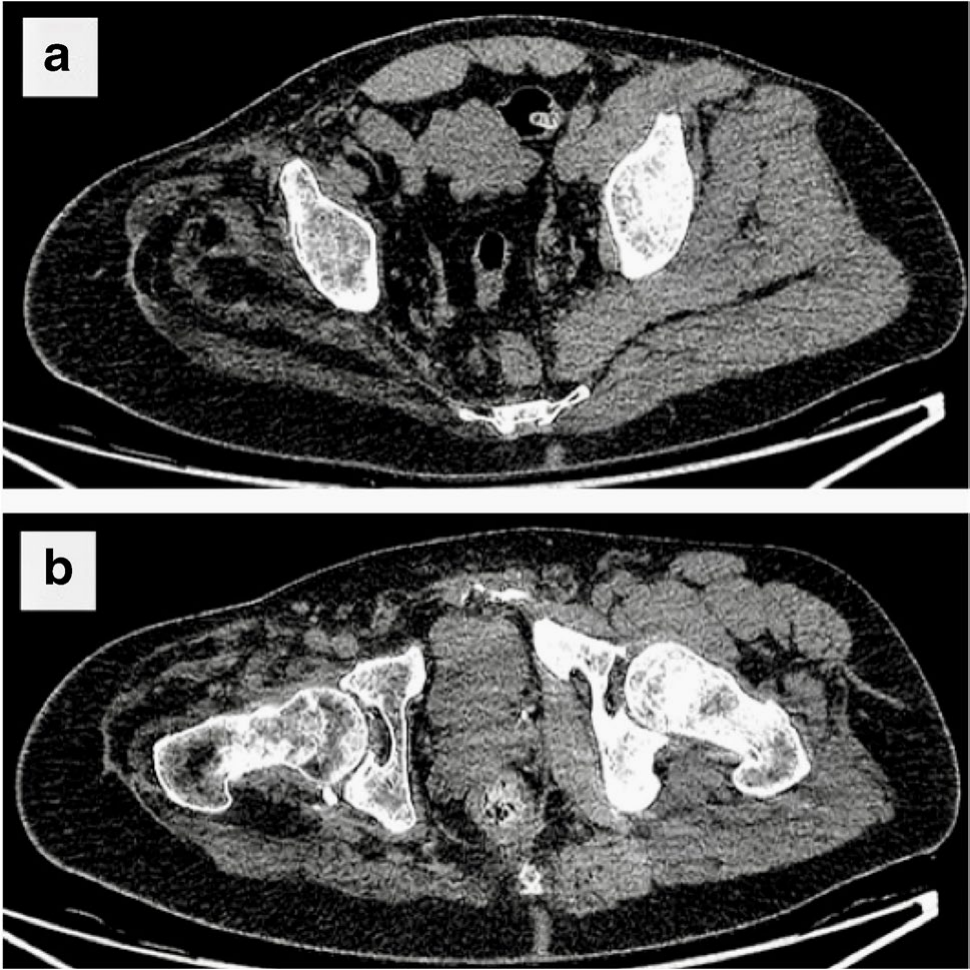
Planning CT images of a patient being evaluated for a right femoral bone-anchored implant. **a**, **b** Severe atrophy of the muscles around the right pelvic girdle. Disuse muscular atrophy is always seen after amputation. However, this degree of advanced atrophy is only seen with the denervation that accompanies a lumbo-sacral plexus injury and may preclude safe implantation with a bone-anchor since the patient may be unable to control their residual limb. In contrast, osteopenia is not an absolute contraindication to implantation since bone density will increase after implantation, as long as the patient (eventually) loads the implant with a prosthesis

Additional information is often available from CT scans that can also be useful to surgeons. For example, patients who have been involved in multi-trauma often suffer from nerve injuries (especially brachial and lumbo-sacral plexus). The degree of muscle wasting in the affected territories of these nerve injuries can be appreciated on CT (Fig. 8). This provides critical information in terms of determining whether the patient is suitable for insertion of a bone-anchor or when planning a targeted muscle reinnervation (TMR) procedure. Disuse osteopenia can also be appreciated on CT although patients will also need to undergo a pre-operative DEXA scan.

### Critical post-operative information for surgeons

Following surgery, patients are reviewed by their surgeon at 3, 6 and 12 months and once a year thereafter for as long as the bone-anchor remains in place. Plain X-rays are obtained at each visit and a repeat CT scan is also obtained at the 12-month time-point. More imaging is obtained if complications arise.

There are four main complications that may be encountered after insertion of a bone-anchor where radiological support is essential. These are the following:

1. Infection—usually localised to the end of the residual bone
2. Aseptic loosening
3. Mechanical fracture of the implant
4. Periprosthetic fracture

**Fig. 9:**
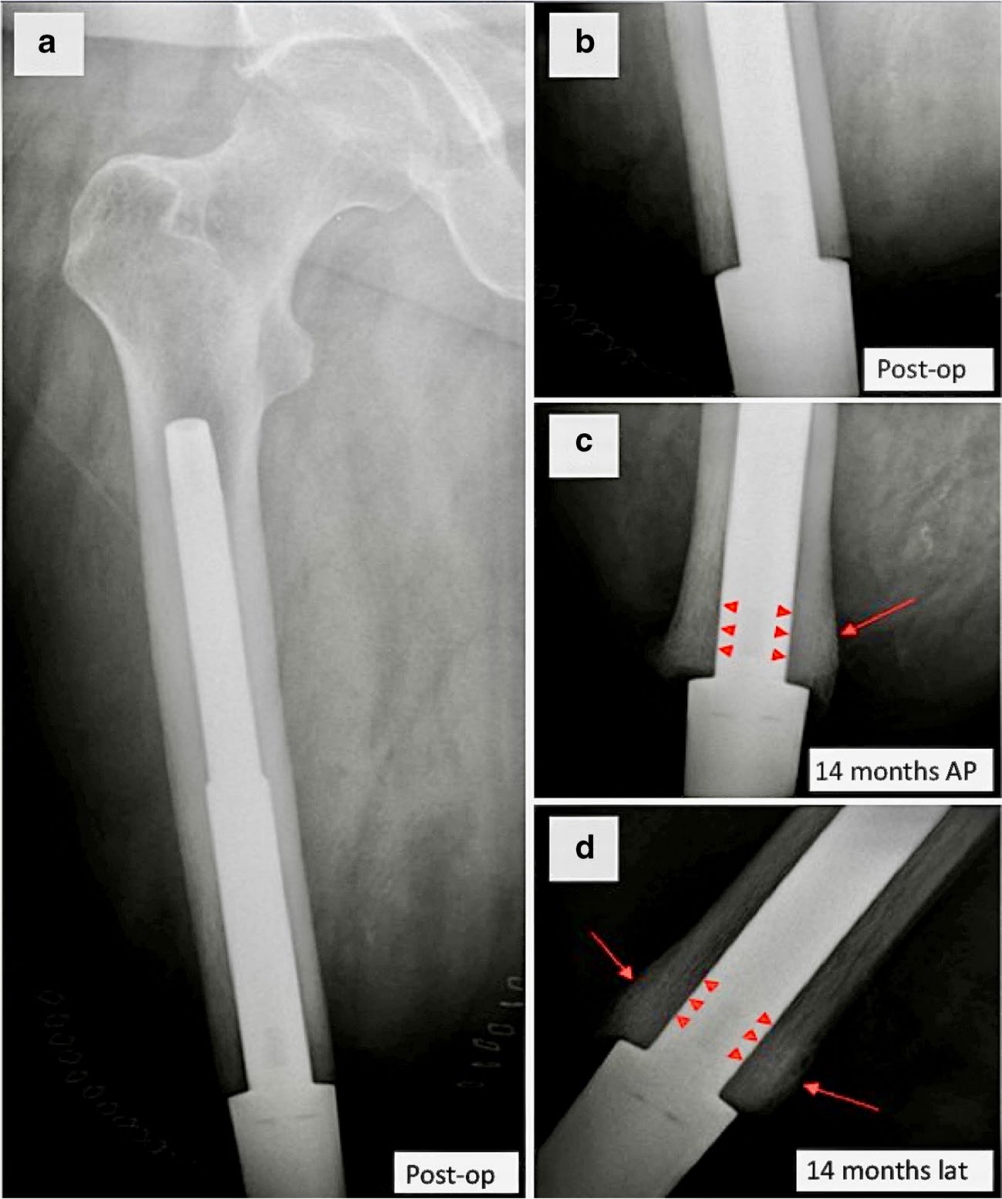
AP radiographs and magnified views showing expected appearances of the implant-bone interface of a transfemoral bone anchor at **a**, **b** the immediate post-operative stage, **c** 7 months and **d** 14 months after insertion. Note thickening of the distal end of the femoral cortex (arrows) and absence of lucency at the bone/implant interface (arrowheads)

In order to put these issues into perspective, it is important for radiologists to understand the normal appearances of the bone following insertion of an OPL bone-anchor (an example is shown in Fig. 9). The critical points to note are the following:

1. Absence of any areas of lucency between the implant and the bone anywhere along its length but especially in the distal 8 cm of the implant (i.e. the most critical part of the implant for osseointegration, through which it will carry the load from the femur).
2. In a lower limb amputee, bone thickening of the distal end of the femur or tibia compared to the X-rays taken immediately post-op. This indicates that the implant is securely anchored to the bone and that the majority of the forces are being carried through the distalmost end of the bone. This thickens in response to loading. This is in keeping with previous radiological studies of OPL bone-anchors [10].
3. The interface between the implant and the bone can also be assessed on CT scans. It is useful to use artefact-reduction algorithms.

**Fig. 10:**
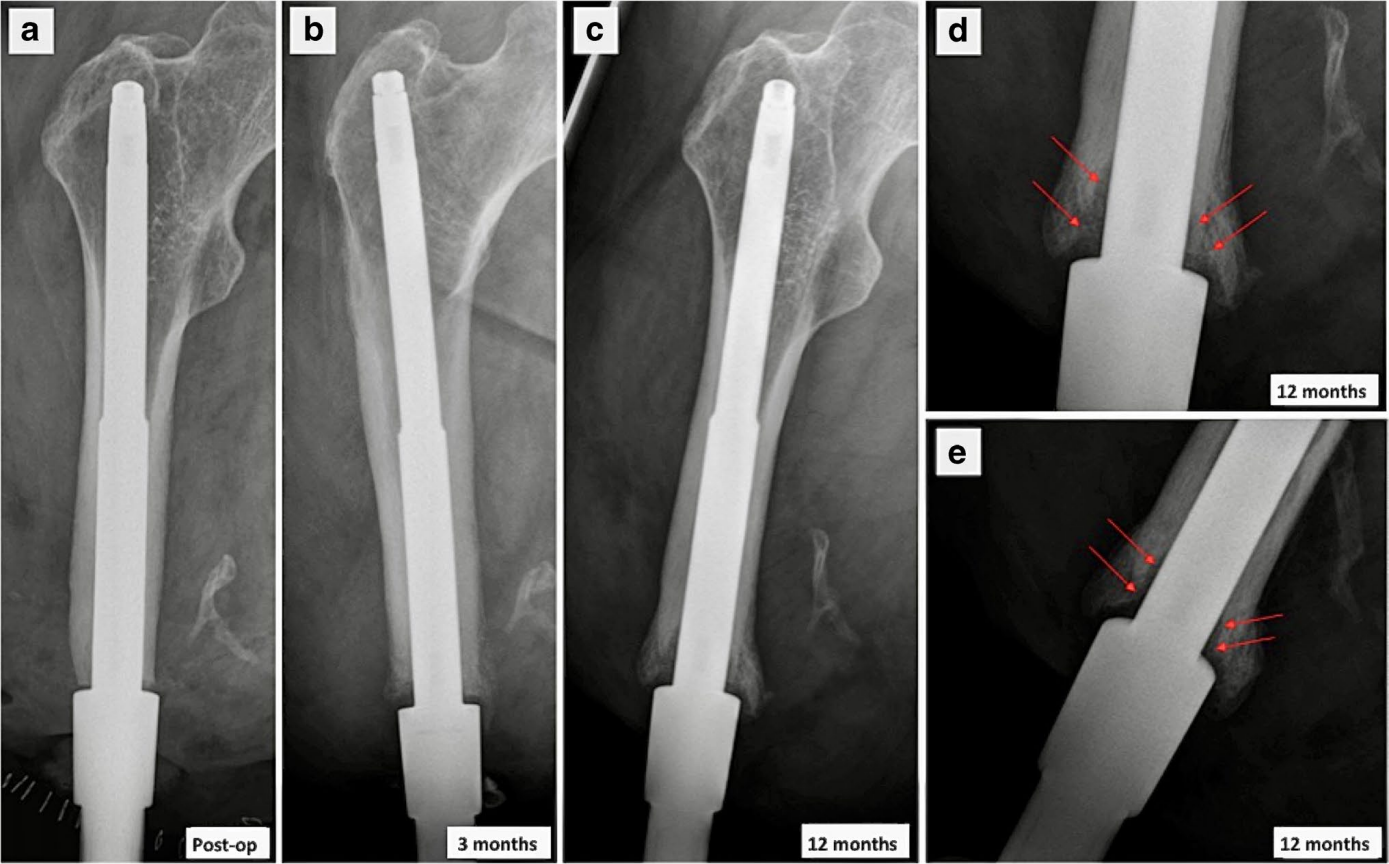
AP radiographs showing a bone-anchor complicated by infection in a transfemoral amputee **a** immediately post-op, **b** 3 months and **c** 12 months following insertion. **d** AP and **e** lateral magnified views of appearances at 12 months. **b**–**e** Serial radiographs show gradual progression of triangular lucent “pockets” causing loss of contact between the implant and inner cortex in the distal femur (arrows)

This contrasts with the X-ray appearances of a patient who developed a localised area of infection in their distal femur (Fig. 10). The patient was diagnosed with ulcerative colitis 6 months after insertion of their bone-anchor and was subsequently treated with prednisolone and infliximab. After 4 weeks of immunosuppression, he developed pain and a bloody discharge from the stump stoma. The key points to note on the X-rays are the loss of contact between the implant and the bone distally, creating lucent triangular-shaped “pockets” in the distal femur. This is where bacteria have proliferated and dissolved the chemical bond between metal and bone that forms the foundation of osseointegration. Since this segment of bone is no longer in contact with the implant, forces from the femur are transferred into the implant more proximally resulting in further bone resorption through the process of “stress-shielding”. The segment of bone where additional forces are being carried remodels in response, resulting in a fusiform thickening of the cortex just above the advancing the edge of the triangular “pocket”. Without intervention, this process would migrate proximally along the entire length of the implant, resulting in complete loosening and loss of the implant.

**Fig. 11:**
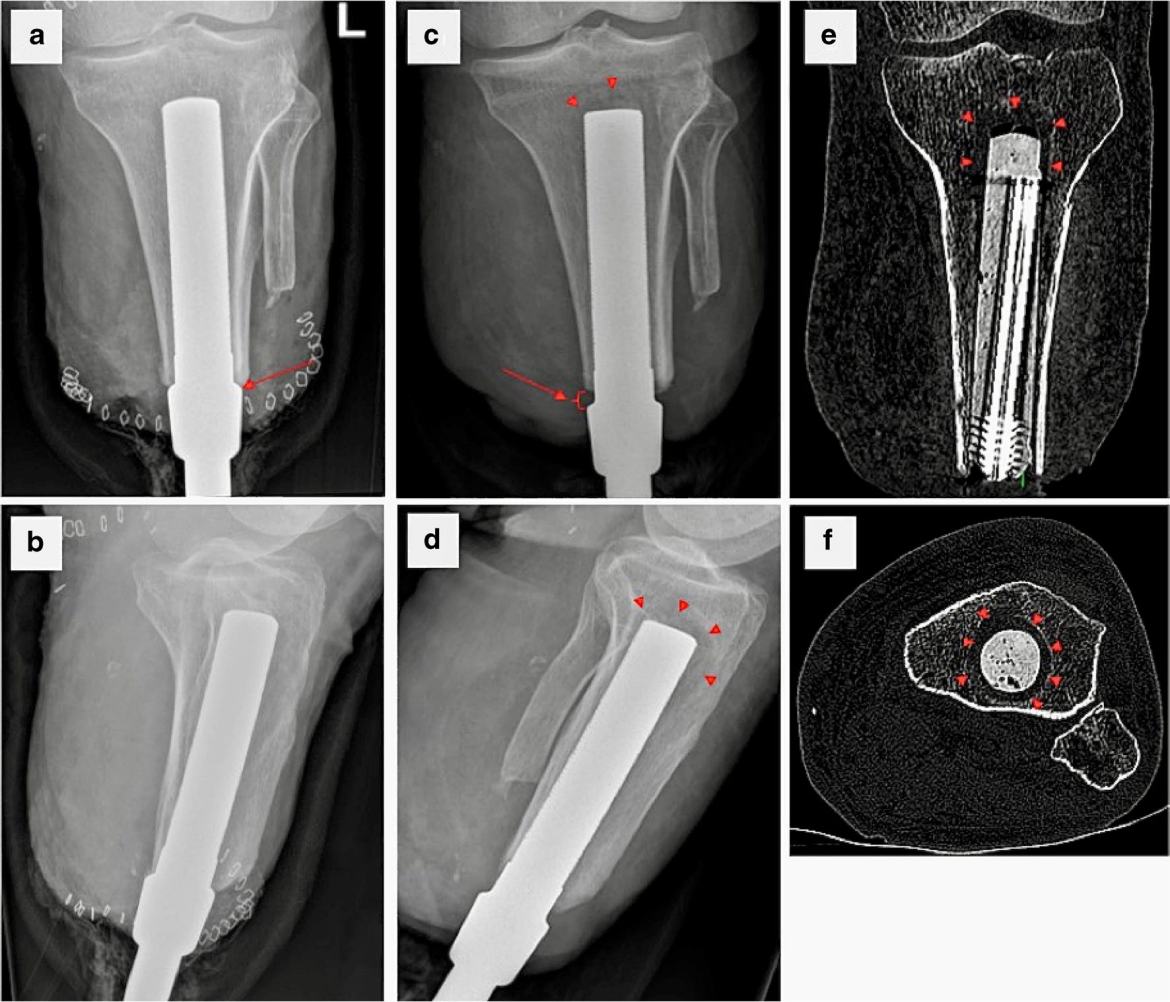
AP and lateral radiographs of a transtibial amputee with bone anchor **a**, **b** immediately post-op and **c**, **d** 6 weeks following insertion, when the patient was the victim of physical assault. **c**, **d** The position of the implant has migrated inferiorly relative to the immediate post-op imaging (arrows) and new lucency surrounding the implant represents crushed trabecular bone that is no longer supporting the implant (arrowheads). **e** Coronal and **f** axial CT images showing the extent of periprosthetic trabecular injury (arrowheads)

Aseptic loosening is another cause of concern with bone-anchors and represents a complete loss of the bone-implant contact which is fundamental to osseointegration. This may be the result of sudden mechanical stress which breaks the bond between metal and bone. Figure 11 shows aseptic loosening in a patient who was the victim of physical assault at 6 weeks post-op whereby the attached prosthesis was violently levered.

Alternatively, loosening can be the result of an indolent infective process caused by low virulence germs which does not result in a symptomatic inflammatory process as seen on the patient in Fig. 10 but which has the same effect on the stability of the implant. Since the outer layer of the OPL implant is composed of a textured layer of titanium that is plasma sprayed onto the core of the implant (also titanium), the outer layer is physically (rather than chemically) bonded to the core. Therefore, since osseointegration also requires that there should be no physical movement between the implant and the surrounding bone, particle disease (caused by microwear of metal fragments from the surface of the implant) is unlikely to be a cause for loosening. From the perspective of the patient, when the amount of bone, to which the implant is chemically bonded by osseointegration, falls below the level needed to tolerate the forces applied to the implant (e.g. by the weight of the prosthesis, walking), the implant will (suddenly) come loose and may spin freely in the bone, meaning that it is no longer able to perform its function as a bone-anchor. This may or may not be accompanied by pain.

Mechanical fracture of the implant is a rare event with the OPL system [5, 9]. More commonly, it is the connector systems that break since they are designed to give way before undue forces sufficient to result in fracture of the bone or mechanical failure are transmitted to the implant. However, when a mechanical fracture of the implant occurs, it will generally be obvious on X-rays and to the patient who is able to perceive the prosthesis through the phenomenon of osseoperception and will immediately sense the loss of integrity [11].

**Fig. 12:**
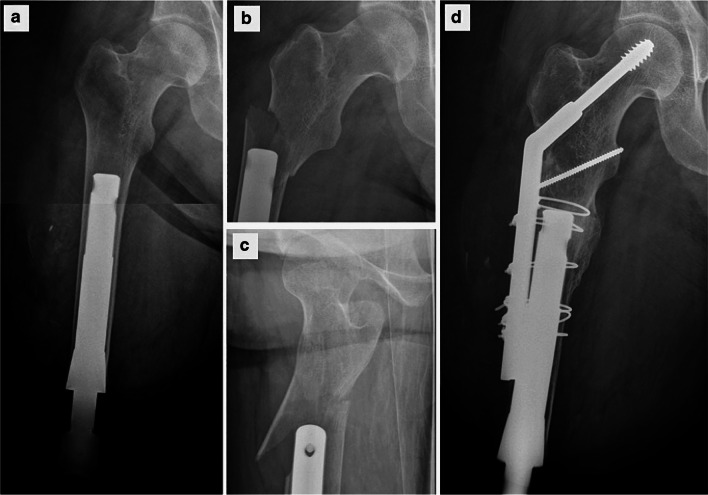
AP radiographs of a transfemoral amputee with bone anchor **a** 4 weeks and **b**, **c** 6 weeks post-op, when he sustained a periprosthetic fracture. **d** The periprosthetic fracture was fixed with a dynamic hip screw and cables

Likewise, periprosthetic fractures are immediately obvious. The diagnosis of a periprosthetic fracture is made on plain X-rays, which are also necessary to follow the progress of healing after fixation. Figure 12 shows a patient who was adapting well to the bone-anchor and sustained an injury following a jump when he felt a “snap” and pain on the hip. The periprosthetic fracture was fixed with a dynamic hip screw and cerclage wires. The patient made a full recovery and continues to use his bone-anchor without difficulty at 4 years after surgery.

## Discussion

Osseointegrated bone-anchors for direct skeletal fixation of a prosthesis are becoming increasingly popular because they can improve the function and quality of life for amputees when compared with a conventional socket-fitted prosthetic solution. A multidisciplinary approach to treatment is essential, and the contributions made by radiology departments are indispensable both before and after surgery. Pre-operatively, radiology is needed to assist in decision-making about the feasibility of surgery and in the selection of an appropriate size of implant and/or the need for any additional surgery. Post-operatively, radiology is needed to monitor outcomes and to look for any potential complications which may arise. This can only happen if radiologists understand how these implants are used and how they behave in the months and years after implantation. Although the data available on the functional outcomes after implantation of an osseointegrated implant are generally good, bony complications are common [2, 5, 6, 8, 9]. Therefore, as with any surgical procedure, radiologists must also understand the unique imaging requirements associated this these implants so that problems can be identified in a timely manner. Radiologists who are involved in the care of this growing cohort of patients must become familiar with the particular characteristics of the most commonly used implant systems so that they can contribute to optimal patient outcomes.

## Data Availability

All of the full resolution images used in this article are freely available for review by the readers using the following link: https://relimbmy.sharepoint.com/personal/norbert_relimb_org/_layouts/15/onedrive.aspx?id=%2Fpersonal%2Fnorbert%5Frelimb%5Forg%2FDocuments%2FResearch%2FRadiology%20for%20bone%2Danchors%2FData%20Availability%20Access%20Folder&ga=1
